# Interhemispheric Callosal Projections Sharpen Frequency Tuning and Enforce Response Fidelity in Primary Auditory Cortex

**DOI:** 10.1523/ENEURO.0256-20.2020

**Published:** 2020-08-14

**Authors:** Bernard J. Slater, Jeffry S. Isaacson

**Affiliations:** Center for Neural Circuits and Behavior and Department of Neurosciences, University of California, San Diego, La Jolla, CA 92093

**Keywords:** callosal, electrophysiology, interneuron, neural circuits, optogenetic, sensory coding

## Abstract

Sensory cortical areas receive glutamatergic callosal projections that link information processing between brain hemispheres. In primary auditory cortex (A1), ipsilateral principal cells from a particular tonotopic region project to neurons in matching frequency space of the contralateral cortex. However, the role of interhemispheric projections in shaping cortical responses to sound and frequency tuning in awake animals is unclear. Here, we use translaminar single-unit recordings and optogenetic approaches to probe how callosal inputs modulate spontaneous and tone-evoked activity in A1 of awake mice. Brief activation of callosal inputs drove either short-latency increases or decreases in firing of individual neurons. Across all cortical layers, the majority of responsive regular spiking (RS) cells received short-latency inhibition, whereas fast spiking (FS) cells were almost exclusively excited. Consistent with the callosal-evoked increases in FS cell activity *in vivo*, brain slice recordings confirmed that parvalbumin (PV)-expressing cells received stronger callosal input than pyramidal cells or other interneuron subtypes. Acute *in vivo* silencing of the contralateral cortex generally increased spontaneous firing across cortical layers and linearly transformed responses to pure tones via both divisive and additive operations. The net effect was a decrease in signal-to-noise ratio for evoked responses and a broadening of frequency tuning curves. Together, these results suggest that callosal input regulates both the salience and tuning sharpness of tone responses in A1 via PV cell-mediated feedforward inhibition.

## Significance Statement

We use *in vitro* intracellular and *in vivo* extracellular recordings to show how interhemispheric projections modulate sensory representations in primary auditory cortex (A1). Callosal projections make preferential input onto parvalbumin (PV)-expressing interneurons, particularly to those in deeper layers. Silencing the contralateral cortex increased principal neuron spontaneous activity and broadened frequency tuning. These results indicate that the primary effect of the interhemispheric projection is to sharpen frequency tuning and enforce the signal-to-noise ratio.

## Introduction

Cortical sensory representations driven by thalamic inputs are strongly influenced by local intracortical circuits and long-range projections including interhemispheric inputs (Cerri et al., 2010; Schmidt et al., 2010; Carrasco et al., 2013, 2015; Li et al., 2013; Lien and Scanziani, 2013; Wunderle et al., 2015; Lee et al., 2019). In most sensory systems, there is an early decussation such that each hemifield of a sensory modality is primarily represented in the contralateral hemisphere of the brain. However, sensory areas for a particular modality in both cortices are linked to each other via interhemispheric projections from axons within the corpus callosum. These long-range, corticocortical projections contact a majority of neurons in both supragranular and infragranular layers (Wise and Jones, 1976; Carr and Sesack, 1998; Petreanu et al., 2007), but their postsynaptic targets and degree of connectivity vary in different sensory cortical areas (Harris et al., 2019). The differences in callosal connectivity with pyramidal cells and local interneurons is reflected in previous studies indicating that activation of callosal inputs can drive excitation and/or inhibition in cortical circuits (Karayannis et al., 2007; Lee et al., 2014; Rock and Apicella, 2015; Anastasiades et al., 2018). Although these studies have begun to characterize the functional properties of interhemispheric cortical projections, how callosal pathways contribute to sensory coding *in vivo* is not well understood.

Unlike the visual and somatosensory cortices where interhemispheric inputs are relegated to hemifield overlap areas (Choudhury et al., 1965; Ebner and Myers, 1965; Hubel and Wiesel, 1967; Conti et al., 1986; Manzoni et al., 1989), callosal inputs are widespread across the tonotopically-organized primary auditory cortex (A1; Code and Winer, 1985, 1986; Hackett and Phillips, 2011). Furthermore, anatomic studies in cats indicate that callosal projections between primary auditory areas are “homotypic”: projections arising from a particular tonotopic region in one cortex map onto the corresponding frequency space within the contralateral cortex (Diamond et al., 1968; Imig and Brugge, 1978; Rouiller et al., 1991; Lee and Winer, 2008). Although less is known regarding the specificity of callosal projections in rodents, homotypic interactions have also been found in anatomic studies of rats (Cipolloni and Peters, 1983; Rüttgers et al., 1990). Although callosal inputs arise from the axons of pyramidal cells in the opposite cortex, this pathway may not simply lead to cortical excitation. Indeed, in anesthetized ferrets, electrical stimulation of callosal inputs caused a variety of effects on sound-evoked firing rates including enhancement, suppression, or a mixture of the two (Kitzes and Doherty, 1994). Furthermore, intracellular recordings in A1 of anesthetized cats found that electrical stimulation in contralateral A1 elicited excitatory postsynaptic potentials that were often followed by inhibitory postsynaptic potentials (Mitani and Shimokouchi, 1985). These findings are consistent with a recent brain slice study indicating that A1 callosal inputs drive strong activation of layer 5 (L5) parvalbumin (PV) cells that mediate feedforward inhibition of pyramidal cells (Rock and Apicella, 2015). Despite these results suggesting a potential inhibitory influence of callosal inputs in auditory processing, removing interhemispheric input in anesthetized cats using cortical cooling reduced sound-evoked activity in contralateral primary cortex (Carrasco et al., 2013). However, anesthesia itself strongly influences spontaneous and sensory-evoked activity in sensory cortex (Harris and Thiele, 2011; Kato et al., 2015), and it is unclear how callosal input modulates A1 sensory processing in the awake state.

Previous studies have probed the contribution of long-range intercortical projections to sensory processing in auditory cortex. For example, stimulation of somatosensory cortex or other cortical areas can alter frequency tuning in auditory cortex neurons by causing a shift in their preferred frequency (Gao and Suga, 2000; Ma and Suga, 2001; Winkowski et al., 2018). Alternatively, other studies have reported that input from visual or motor cortices can suppress activity in auditory cortex principal cells (Bizley et al., 2007; Kayser et al., 2008; Schneider et al., 2018).

In this study, we use linear silicon probes spanning cortical layers to record spontaneous and tone-evoked single-unit activity in A1 of awake, head-fixed mice. We express channelrhodopsin-2 (ChR2) in callosal fibers to study how their local activation modulates activity *in vivo* and identify the local circuits driven by callosal input in brain slice recordings. Finally, we use ChR2 in GABAergic interneurons to acutely suppress activity in one hemisphere while recording tone-evoked responses in contralateral A1 to show how the callosal pathway modulates cortical sensory processing. We find that callosal input drives strong feedforward inhibition of principal cells in A1, likely as a result of stronger excitation onto PV-expressing interneurons. Furthermore, callosal projections mediate both a sharpening in frequency tuning as well as enforcement of signal-to-noise ratio.

## Materials and Methods

Mice (8–16 weeks old for *in vivo* recordings, three to five weeks old for *in vitro* recordings) of either sex, Emx1-Cre (The Jackson Laboratory no. 05638), Gad2-Cre (The Jackson Laboratory no. 019022), PV-cre (The Jackson Laboratory no. 017320), SOM-Cre (The Jackson Laboratory no. 010708), vasoactive intestinal polypeptide (VIP)-cre (The Jackson Laboratory no. 010908), tdTomato reporter (Ai14, The Jackson Laboratory no. 00914), and wild-type C57Bl6 mice were housed with a 12/12 h reversed light cycle. *In vivo* experiments were performed during the dark period. All procedures were in accordance with protocols approved by the University of California, San Diego Institutional Animal Care and Use Committee and guidelines of the National Institutes of Health.

### Surgical preparation

For *in vivo* electrophysiology experiments, two to three weeks before head-bar implantation and habituation to head fixation, mice were anesthetized with isoflurane (2%), and the brain area corresponding to A1 identified by intrinsic imaging (Kato et al., 2015, 2017). Viruses [AAV9-hSyn-hChR2(H134R)-eYFP-WPRE-hGH for activation of callosal terminals or AAV9-Ef1α-DIO-hChR2(h134R)-YFP-WPRE-hGHpA (AAV-FLEX-ChR2; Atasoy et al., 2008) for cre-dependent expression in Gad2-cre mice, UPenn] were injected (50 nl) using beveled pipettes (Nanoject II, Drummond) at three sites spanning A1 at depths of 0.25–0.75 mm. After injections, mice received dexamethasone (2 mg/kg), buprenorphine (0.1 mg/kg), and baytril (10 mg/kg) before returning to their home cage. Two to three days before *in vivo* recording, a head bar was implanted, and A1, contralateral to the virus injection, was identified using intrinsic imaging. For ipsilateral silencing experiments, the previous intrinsic imaging for virus injections was used.

For *in vitro* recordings, neonatal mice (postnatal day 0–2) were anaesthetized by hypothermia and secured in a molded platform. AAV9-hSyn-hChR2(H134R)-eYFP-WPRE-hGH was injected at three locations containing the rostral-caudal axis of the auditory cortex identified by landmarks including the superficial temporal vein (Kato et al., 2017). At each site, injection was performed at three depths (600, 500, and 400 μm deep from the skin surface, 23 nl/site). Neonatal virus injection led to widespread expression of ChR2 in A1 and non-A1. Brain slices were prepared from mice 21–35 d old. Briefly, mice were anesthetized with isoflurane (2%), and the was brain removed into ice-cold artificial CSF (aCSF) containing the following: 83 mm NaCl, 2.5 mm KCl_2_, 0.5 mm CaCl_2_, 3.3 mm MgSO_4_, 1 mm NaH_2_PO_4_, 26.2 mm NaHCO_3_, 22 mm glucose, and 72 mm sucrose, equilibrated with 95% O_2_ and 5% CO_2_. Coronal slices (400 μm thick) from the cortex contralateral to the virus injection site were cut using a vibrating slicer (DSK). Slices were selected to contain A1 based on landmarks including the rhinal fissure and shape of the hippocampal formation (2.18–2.92 from bregma; Franklin and Paxinos, 2008). Although *in vitro* recordings were targeted to A1 based on these landmarks, we cannot exclude the possibility that some recordings were obtained from neighboring, non-A1.

### Extracellular recordings

A 32-channel (Neuronexus) or 64-channel (Cambridge Neurotech) silicon probe was used for extracellular recordings. Signals were recorded using an Intan RHD2000 and digitized at 20 kHz using Open Ephys (Siegle et al., 2017). Spikes were sorted using Kilosort (Pachitariu et al., 2016), followed by manual curation in phy (Rossant et al., 2016) to obtain single units used for analyses. Cells were excluded from analysis if they did not maintain consistent firing and amplitude throughout recording, and a firing rate of at least 1 Hz. The probe was coated in DiI to verify probe track for depth of recording as well as recording location. Current source density (Pettersen et al., 2006) coupled with anatomic verification of probe track was used to identify laminar single-unit locations. For all recordings spike waveforms were obtained from the lead with the largest amplitude template, these were then averaged to obtain an average spike waveform. Units were classified as fast spiking (FS) if their average spike waveform had a trough to peak time of <300 μs and a full width at half maximum of <125 μs.

A fiber-coupled LED (470 nm, 20 mW, 0.4 mm fiber, 0.48 N.A., Thorlabs) was positioned within 1–2 mm of the exposed cortical surface for activating ChR2-expressing callosal fibers or ipsilateral cortical silencing. For experiments using contralateral silencing, the skull over the virus-expressing auditory cortex was exposed and covered with cyanoacrylate glue (to improve translucency) before the LED fiber was positioned at the skull surface. Callosal fiber activation was achieved using a single 5 ms flash (20 mW). For cortical silencing in Gad2-cre mice expressing ChR2, we used a train of 10-ms light pulses (510 ms, 20 Hz, 20 mW) to activate inhibitory interneurons.

Mice were anesthetized with isoflurane (2%) immediately before recording and the ear canal ipsilateral to the recorded cortex was occluded with cyanoacrylate glue to minimize bilateral auditory input. A well filled with aCSF (142 mm NaCl, 5 mm KCl, 10 mm glucose, 10 mm HEPES, 3.1 mm CaCl_2_, and 1.3 mm MgCl_2_; pH 7.4, 310 mOsm) was constructed around the recording site, and a small (<0.3 mm) craniotomy was performed through thinned skull. Mice recovered for >1 h before the start of recording. Pure tones (250 ms duration) logarithmically spaced between 4 and 60 kHz (60-dB SPL, 5 ms rise/fall, 1-s intertrial interval) were delivered via a calibrated free-field speaker (ES1, TDT) directed to the left ear. Tones were generated by software (BControl; http://brodylab.org) running on MATLAB (MathWorks) communicating with a real-time system (RTLinux). Tone frequencies were presented in a pseudo-random fashion and LED illumination was delivered on interleaved trials.

### *In vitro* electrophysiology

Patch-clamp recordings were performed using an upright microscope, 40× objective, and DIC optics. Recordings were made using a Multiclamp 700A amplifier (Molecular Devices), digitized at 20 kHz, and acquired and analyzed using AxographX software. For voltage-clamp recordings, pipettes (3–5 MΩ) contained the following: 130 mm D-gluconic acid, 130 mm CsOH, 5 mm NaCl, 10 mm HEPES, 10 mm EGTA, 12 mm phosphocreatine, 3 mm Mg-ATP, and 0.2 mm Na-GTP; pH 7.3. Series resistance was routinely <20 MΩ and continuously monitored. LED illumination (470 nm, Thorlabs) was delivered through the microscope objective.

### Analysis of *in vivo* data

For presentation of pooled neuronal responses, firing rates were normalized to the average baseline firing rate of each neuron 250 ms before the LED period. The analysis window for callosal terminal excitation was 10 ms from LED onset to capture both the initial excitation and recurrent inhibition. In contralateral A1 silencing experiments, the window for analysis was a 250-ms time period that started 250 ms after LED onset. All statistical tests were two sided and used a significance level of 0.05 (corrected for multiple comparisons where noted). Units were considered significantly modulated by the LED if the mean firing rate during the analysis window was different from that of the baseline period as determined by a Wilcoxon sign-rank test α = 0.05. Modulation index was calculated as [(mean firing rate in analysis window) – (mean firing rate during baseline period)]/[(mean firing rate in analysis window) + (mean firing rate during baseline period)]. Average modulation of units was tested for significance using a one sample *t* test.

Sound responses were determined as significant at a given frequency if *p* < 0.05 for a Wilcoxon rank-sum test of firing rate over 250 ms starting 10 ms after sound onset as compared with the same time period during interleaved trials with no tones (blank trials). A Holm–Bonferroni correction was used for multiple comparisons. Units were considered sound responsive if they responded to at least one tone frequency. Unit responses to a given frequency were averaged and these average responses were fit with a linear polynomial. RS units were included in analysis if they were sound responsive and had a linear fit with *r*^2^ > 0.25. Slope significance was determined using a 95% confidence interval for the linear fit, slopes were considered significantly modulated either divisively or multiplicatively if the upper bound was <1 or the lower bound was >1, respectively. Intercept significance was determined using a 95% confidence interval for the linear fit, intercepts were considered significantly modulated in either an additive or subtractive fashion where lower bound was >0 or the upper bound was <0, respectively. The discriminability index, *d*′, was calculated for the average of every LED modulated tone response as (mean Spikes_sound_ − mean Spikes_spontaneous_)/√[0.5 × (σ^2^_sound_ + σ^2^_spontaneous_)]. Tone responses for a given unit were excluded if their tone response versus spontaneous firing rate *z* score was <2. The *d*′ values are presented as the mean of *d*′ values for a given unit. To generate a frequency tuning curves, individual unit responses were averaged at each frequency. The responses were then centered to the best frequency (BF) chosen as the frequency which had the strongest tone response in the control condition for each unit. Significant modulation at each frequency by cortical inactivation was determined using a paired *t* test followed by a Holm–Bonferroni correction for multiple comparisons.

## Results

**Figure 1.**
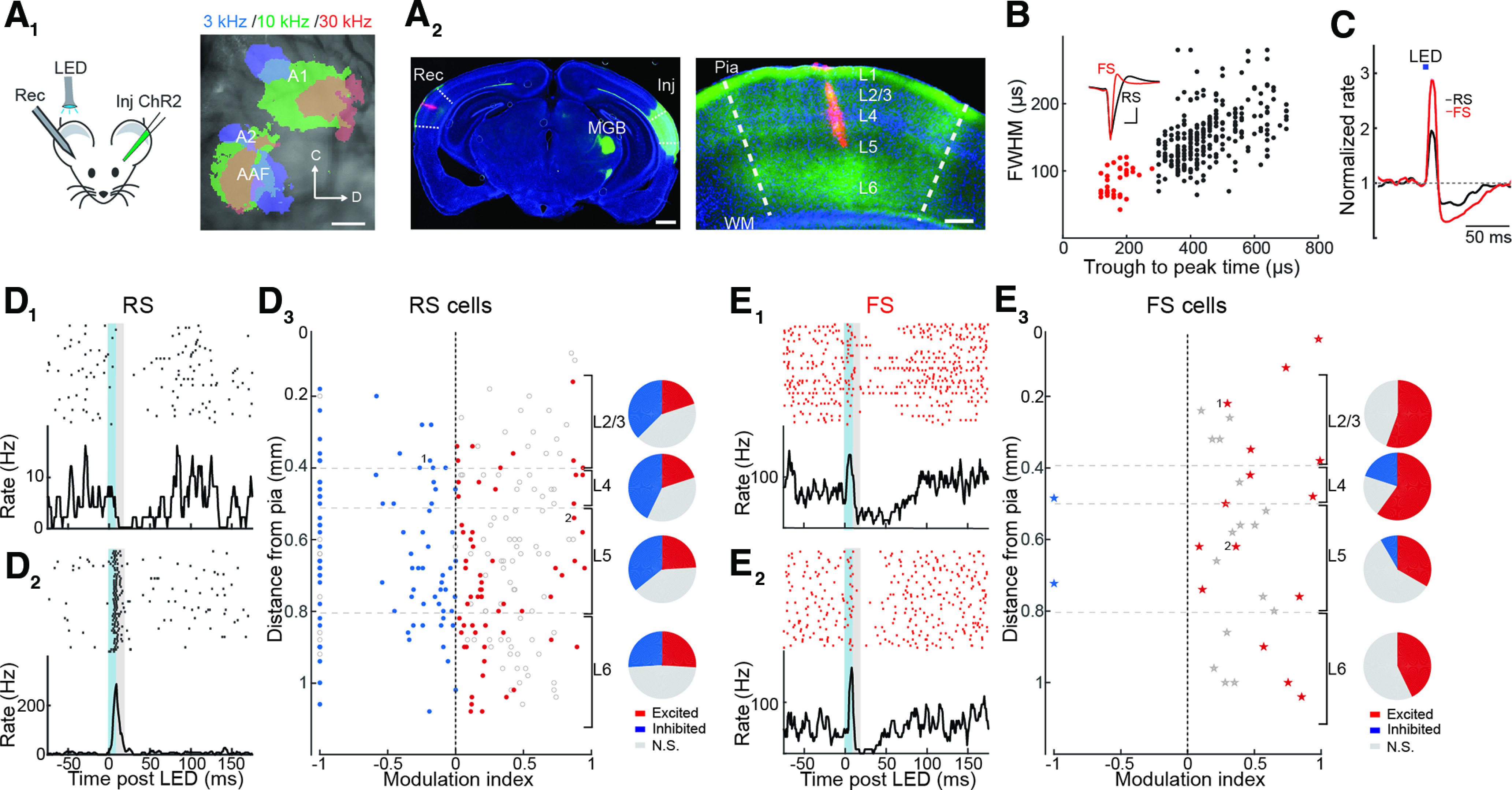
Optogenetic activation of cortical callosal inputs evokes excitation and inhibition in A1 of awake mice. ***A_1_***, left, Experiment schematic, wild-type C57Bl6 mice. Right, Intrinsic imaging showing responses to 3-, 10-, and 30-kHz pure tones overlaid on an image of the vasculature. Areas indicated are A1, AAF, and A2. Scale bar = 500 μm. ***A_2_***, left, Coronal section showing ChR2 expression (green) within A1 of the injected left hemisphere (Inj) and DiI-labeled recording electrode tract (red) in contralateral A1 (Rec). Dense ChR2 expression is also present in the MGB of the injected hemisphere. Scale bar = 1 mm. Right, Blow-up of recording site in the right hemisphere shows expression of ChR2-expressing fibers throughout all cortical layers. WM = white matter. Scale bar = 250 μm. Dashed lines show A1 border inferred from the same coronal planes according to Franklin and Paxinos (2008). ***B***, FS (red) and RS (black) units are identified by plotting spike trough to peak time versus full width at half maximum (FWHM). Inset, Average waveforms of FS and RS units. Scale bar = 250 μs, 20 μV. ***C***, Average normalized peristimulus time histogram (PSTH) of RS (black) and FS (red) units shows that brief LED illumination (bar) drives a transient increase followed by a decrease in firing rate. ***D***, Activation of callosal inputs increases activity of some RS cells, but inhibition is more widespread. ***D_1_***, Individual RS unit spike raster and PSTH showing that ChR2 activation of callosal fibers (blue shading) inhibits firing. Gray shading indicates measurement period used to calculate modulation index. ***D_2_***, RS unit strongly activated by callosal input. ***D_3_***, left, Modulation index of units significantly activated (red) or inhibited (blue) across all layers. Open circles indicate units without significant effect and points marked 1 and 2 represent units in ***D_1_***, ***D_2_***, respectively. Right, Pie charts indicate proportion of units excited (red), inhibited (blue), or not significantly modulated (gray) in each layer. ***E***, Activation of callosal inputs activates FS cells across all layers. Two representative FS units are plotted in ***E_1_***, ***E_2_***. ***E_3_***, Modulation index of FS units across all cell layers are illustrated as for RS cells in ***D_3_***.

We first studied how local activation of callosal projections modulates cortical excitability by targeting injection of adeno-associated virus (AAV) expressing ChR2 to A1 of the left hemisphere (Fig. 1*A*) in wild-type C57Bl6 mice. Dense expression of ChR2 in fibers within the left medial geniculate body (MGB) confirmed that injections targeted auditory cortex (Fig. 1*A_2_*). Although we targeted A1 for virus injection, other auditory cortical areas [i.e., anterior auditory field (AAF) and non-A1] are likely to also be labeled. We inserted linear silicon electrodes in A1 of the right hemisphere to monitor single-unit activity in the awake state. *Post hoc* analysis of probe recording sites revealed callosal ChR2-expressing fibers distributed across all layers of A1 (Fig. 1*A_2_*). Trough to peak time and full width at half maximum of spike waveforms (Fig. 1*B*) were used to classify single units as regular spiking (RS; principal cells) or FS (presumptive PV-expressing interneurons).

We used brief (5 ms) LED illumination (470 nm) of the recording site to activate callosal inputs. On average, callosal stimulation caused a biphasic response in both RS (*n* = 264) and FS (*n* = 33, *n* = 7 mice) cells: a rapid increase in firing rate followed by a decrease in firing that returned to baseline over 50–100 ms (Fig. 1*C*). However, individual RS cells in the same experiments responded quite differently from each other: some cells were transiently excited by callosal stimulation, while others were exclusively inhibited (Fig. 1*D_1_*,*D_2_*). We used a modulation index (Materials and Methods) to quantify early changes in firing (within 10 ms of callosal LED stimulation). We found that RS cells were more likely to be significantly inhibited than excited (*p* < 0.05, sign test; Fig. 1*D_3_*) in layers 2/3 (L2/3), 4 (L4), and L5, while cells were equally likely to be excited or inhibited in layer 6 [L6; inhibited vs excited, L2/3: 38 vs 20% (*n* = 23 responding units), L4: 43 vs 20% (*n* = 22), L5: 36 vs 24% (*n* = 67), L6: 26% for each (*n* = 40)]. In contrast, FS cells were much more likely to be significantly excited than inhibited by callosal stimulation across all layers (*n* = 15 excited vs 2 inhibited; Fig. 1*E*). Together, these *in vivo* results indicate that while a subset of pyramidal cells are directly excited by callosal inputs, interhemispheric projections cause a widespread suppression of pyramidal cell activity. The rapid increase in FS cell firing evoked by activation of callosal inputs suggests that principal cell suppression arises from PV cell-mediated feedforward inhibition.

**Figure 2.**
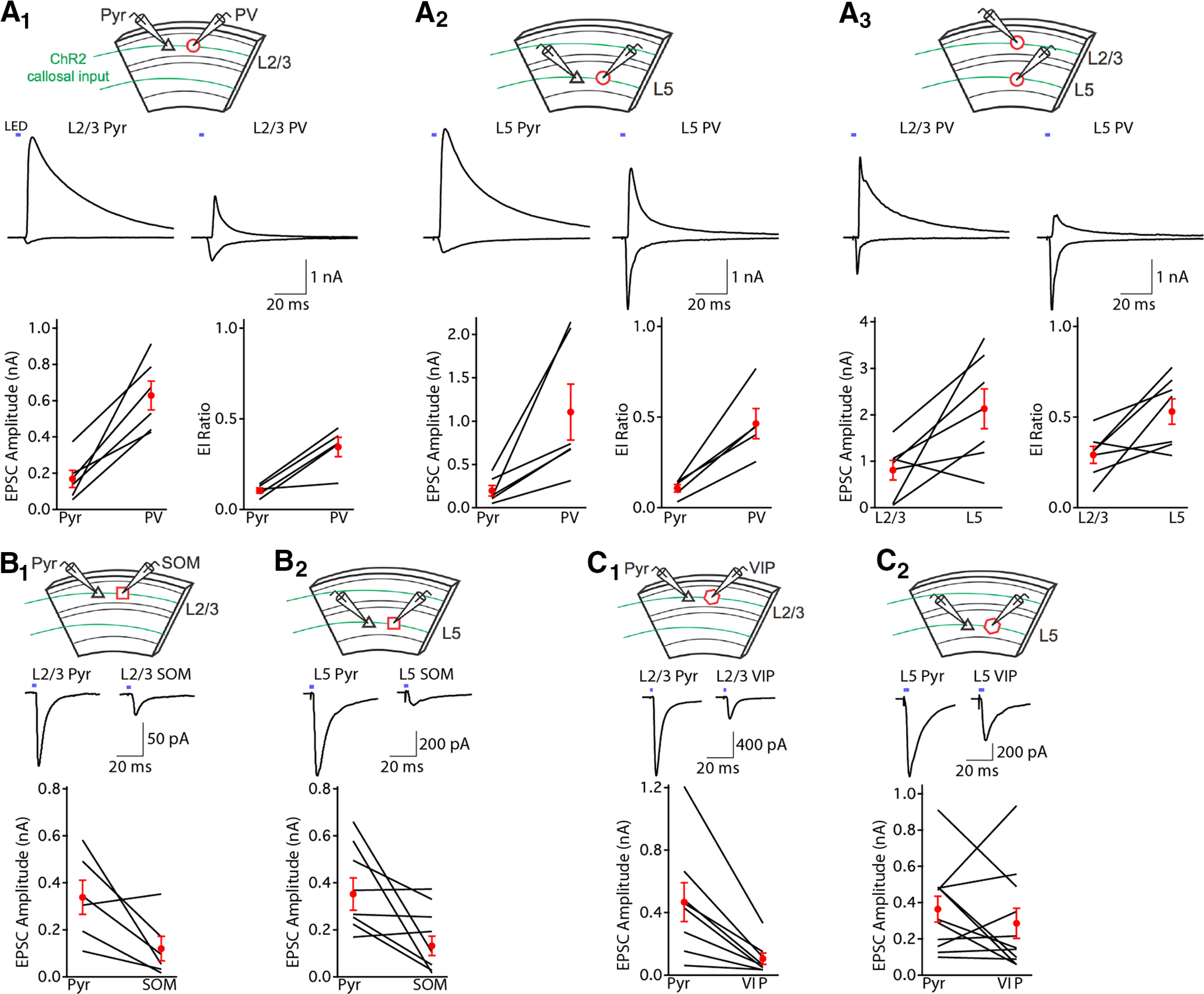
Cortical callosal inputs preferentially excite PV cells and drive strong feedforward inhibition. ***A_1_***, L2/3 PV cells receive stronger callosal fiber-evoked EPSCs and have a larger E/I ratio than L2/3 pyramidal cells. Top, Recording configuration. Middle, Simultaneous voltage-clamp recording of L2/3 pyramidal cell (Pyr) and PV cell showing EPSCs (inward currents, −70 mV) and IPSCs (outward currents, +10 mV) evoked by brief LED illumination (blue bars) of ChR2-expressing callosal fibers. Bottom, Summary of EPSC peak amplitudes and E/I ratios for recorded pairs. Black lines, individual cell pairs. Red circles, mean ±SEM. ***A_2_***, L5 PV cells receive stronger callosal fiber-evoked EPSCs and have a larger E/I ratio than L5 pyramidal cells. ***A_3_***, L5 PV cells receive stronger callosal fiber-evoked EPSCs and have a larger E/I ratio than L2/3 PV cells. ***B***, SOM cells in L2/3 (***B_1_***) and L5 (***B_2_***) receive weaker callosal fiber-evoked EPSCs than neighboring pyramidal cells. ***C***, VIP cells in L2/3 (***C_1_***) receive weaker callosal fiber-evoked EPSCs than neighboring pyramidal cells. The strength of callosal input-evoked EPSCs in L5 VIP cells (***C_2_***) and pyramidal cells are similar.

We next used voltage-clamp recordings in brain slices to better understand the layer and cell type specificity of callosal input. We first examined the relative strength of callosal input onto PV and pyramidal cells. PV-Cre mice were crossed to a td-Tomato reporter line (Ai14) to target whole-cell recordings of visually identified PV cells. Neonatal virus injection in the left auditory cortex was used to drive expression of ChR2 in callosal fibers of the contralateral (right) auditory cortex. We measured responses using simultaneously recorded pairs of PV and pyramidal cells (Pyr) from L2/3 of A1 contralateral to the injection (Fig. 2*A_1_*). At −70 mV (near the reversal potential for GABAergic inhibition), brief LED illumination (470 nm, 2–4 ms) elicited EPSCs that were much larger in PV than pyramidal cells (peak EPSC amplitude PV = 628 ± 80 pA, Pyr = 168 ± 50 pA, *n* = 6 pairs, *p* = 0.003, paired *t* test). Depolarization to +10 mV (near the reversal potential for glutamatergic excitation), revealed callosal input-evoked IPSCs in both cell types. IPSCs always followed EPSCs with a brief delay in pyramidal and PV cells (average latency 2.13 ± 0.51 ms, *n* = 8, and 1.81 ± 0.2 ms, *n* = 10, respectively) indicating that inhibition was evoked indirectly by callosal input in a feedforward fashion (Isaacson and Scanziani, 2011). The ratio of excitation to inhibition (E/I ratio) was also markedly smaller in pyramidal than PV cells in L2/3 (0.11 ± 0.01 and 0.33 ± 0.06, respectively, *n* = 5 pairs, *p* = 0.01, paired *t* test). Similarly, recordings in pairs of L5 pyramidal and PV cells revealed stronger callosal excitation of PV cells (peak EPSC amplitude PV = 1105 ± 324 pA, Pyr = 197 ± 60 pA, *n* = 6 pairs, *p* = 0.03, paired *t* test; Fig. 2*A_2_*), a smaller pyramidal cell E/I ratio (ratio PV = 0.46 ± 0.08, Pyr = 0.11 ± 0.02, *n* = 5 pairs, *p* = 0.007, paired *t* test), and disynaptic IPSC latency (PV = 1.48 ± 0.07 ms, *n* = 10, Pyr = 1.08 ± 0.11 ms, *n* = 5). Interestingly, paired recordings of L2/3 and L5 PV cells indicated that PV cells in deeper cortical layers receive more callosal excitation (peak EPSC amplitude L2/3 = 0.81 ± 0.21 nA, L5 = 2.13 ± 0.43 nA, *n* = 7 pairs, *p* = 0.03, paired *t* test; Fig. 2*A_3_*) and had a higher E/I ratio (L2/3 = 0.29 ± 0.05, L5 = 0.54 ± 0.07, *n* = 7 pairs, *p* = 0.03, paired *t* test). These findings indicate that callosal projections drive stronger excitation of PV cells than pyramidal cells in both infragranular and supragranular layers. Furthermore, activation of callosal input drives strong feedforward inhibition of principal cells in A1.

Are PV cells unique or do all classes of interneurons receive stronger callosal input than pyramidal cells? To address this, we recorded callosal input-evoked EPSCs onto pairs of pyramidal cells and td-Tomato-labeled somatostatin (SOM)-expressing or vasoactive intestinal polypeptide (VIP)-expressing interneurons using SOM-Cre and VIP-Cre mice. Activation of ChR2-expressing callosal inputs evoked EPSCs that were markedly weaker in SOM cells compared with pyramidal cells in both L2/3 (peak EPSC amplitude SOM = 120 ± 52 pA, Pyr = 338 ± 73 pA, *n* = 6 pairs, *p* = 0.04, paired *t* test; Fig. 2*B_1_*) and L5 (SOM = 132 ± 41 pA, Pyr = 352 ± 69 pA, *n* = 8 pairs, *p* = 0.03, paired *t* test; Fig. 2*B_2_*). Callosal EPSCs were much weaker in VIP cells compared with pyramidal cells in L2/3 (peak EPSC amplitude VIP = 105 ± 36 pA, Pyr = 467 ± 126 pA, *n* = 8 pairs, *p* = 0.006, paired *t* test; Fig. 2*C_1_*), while responses were roughly similar in L5 (VIP = 285 ± 83 pA, Pyr = 364 ± 71 pA, *n* = 11 pairs, *p* = 0.37, paired *t* test; Fig. 2*C_2_*). The relatively weak callosal-evoked EPSCs in SOM and VIP interneurons suggests that they are not a major target of interhemispheric input.

**Figure 3.**
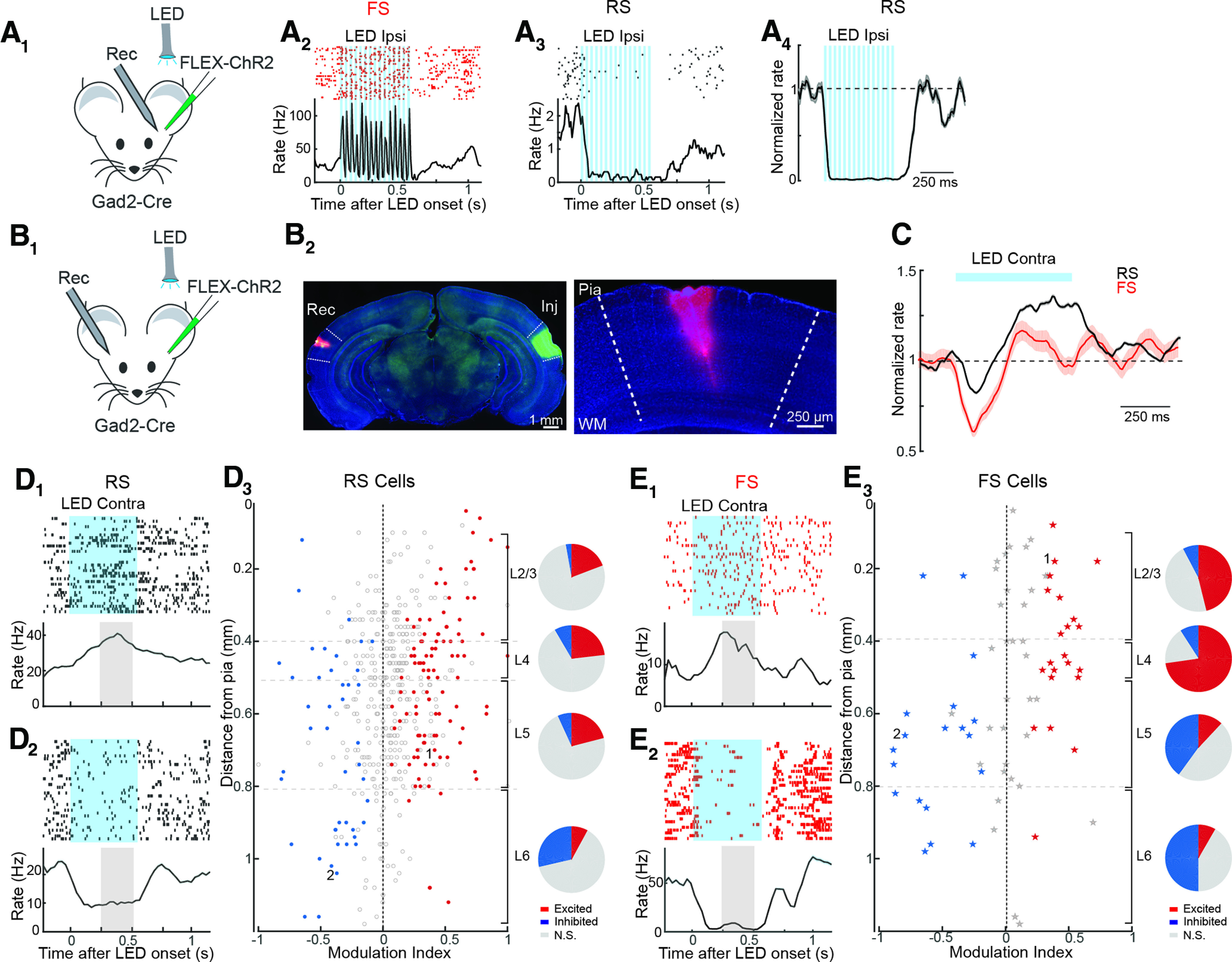
Acute optogenetic silencing of interhemispheric cortical input causes a sustained increase in spontaneous activity in most layers of A1. ***A***, Local activation of ChR2-expressing interneurons silences RS cell activity. ***A_1_***, Recording configuration. ***A_2_***, Spike raster (top) and peristimulus time histogram (PSTH; bottom) show strong activation of a representative FS unit by an ipsilateral LED pulse train (blue bars). ***A_3_***, Spike raster (top) and PSTH (bottom) show strong suppression of simultaneously recorded RS unit. ***A_4_***, Summary of ipsilateral LED-evoked suppression of RS activity (*n* = 34 units, 2 mice). ***B***, Activation of ChR2-expressing interneurons in one hemisphere leads to transient inhibition followed by excitation in contralateral A1. ***B_1_***, Recording configuration. ***B_2_***, left, Coronal section showing ChR2 expression (green) within A1 of the injected left hemisphere (Inj) and DiI-labeled recording electrode tract (red) in contralateral A1 (Rec). Right, Blow-up of recording site. WM = white matter. ***C***, Average normalized PSTH of RS (black) and FS (red) units shows that sustained LED illumination (bar) drives transient decrease and sustained increase in firing. Shading, ±SEM ***D***, Inactivation of A1 causes sustained increase in activity of RS units in layers 1–5 of contralateral A1. ***D_1_***, Individual L5 RS unit spike raster and PSTH showing that silencing contralateral A1 (blue shading) enhances firing. Gray shading indicates measurement period used to calculate modulation index. ***D_2_***, L6 RS unit with sustained suppression during silencing of contralateral A1. ***D_3_***, left, Modulation index of units significantly activated (red) or inhibited (blue) across all layers. Open circles indicate units without significant effect and cells marked 1 and 2 represent units in ***D_1_***, ***D_2_***, respectively. Right, Pie charts indicate proportion of units excited (red), inhibited (blue), or not significantly modulated (gray) in each layer. ***E***, Silencing contralateral A1 causes a rapid and sustained decrease in firing in deep layer FS cells, as well as a sustained firing increase in upper layer FS cells. Representative L2/3 and L5 FS unit are plotted in ***E_1_***, ***E_2_***, respectively. ***E_3_***, Modulation index of FS units across all cell layers are illustrated as in ***D_3_***.

To directly examine the functional role of interhemispheric input *in vivo*, we recorded from A1 in awake mice while optogenetically suppressing activity in the contralateral auditory cortex. We injected AAV-FLEX-ChR2 in the left cortex of Gad2-Cre mice to express ChR2 in GABAergic interneurons (Fig. 3*A_1_*). Recordings in the injected cortex confirmed that LED illumination (20-Hz train of 10-ms pulses) drove firing of FS cells (Fig. 3*A_2_*), while RS cell activity was largely abolished (Fig. 3*A_3_*,*A_4_*). We next monitored spontaneous activity in A1 of the right hemisphere while silencing contralateral A1 (Fig. 3*B_1_*). Although it has been suggested that GABAergic interneurons in auditory cortex can make interhemispheric projections (Rock et al., 2018), we did not observe ChR2-expressing fibers in A1 contralateral to the AAV-injected cortex (Fig. 3*B_2_*). On average, silencing A1 in the left hemisphere caused a transient decrease in firing followed by an increase in activity in RS and FS cells in contralateral, right A1 (*n* = 494 RS, 76 FS, *n* = 19 mice; Fig. 3*B_3_*). However, individual cells responded differently to contralateral silencing depending on cortical layer. LED-responsive RS cells in L2/3, L4, and L5 primarily increased their firing during cortical silencing (excited vs inhibited: 21% vs 6%, *n* = 90 responding units), while L6 RS cells were typically inhibited (excited vs inhibited: 8% vs 29%, *n* = 23 responding units). Similarly, FS cells in L2/3 and L4 were primarily excited during cortical silencing (excited vs inhibited: 49% vs 24%, *n* = 31 responding units) while those in L5 and L6 were more likely to be suppressed (excited vs inhibited: 11% vs 43%, *n* = 20 responding units). These results indicate that spontaneous firing in L6 RS cells and deep layer FS cells is dependent on callosal input. The increase in firing in upper layers during cortical silencing is likely to reflect network effects associated with the withdrawal of deep layer RS and FS cell activity.

**Figure 4.**
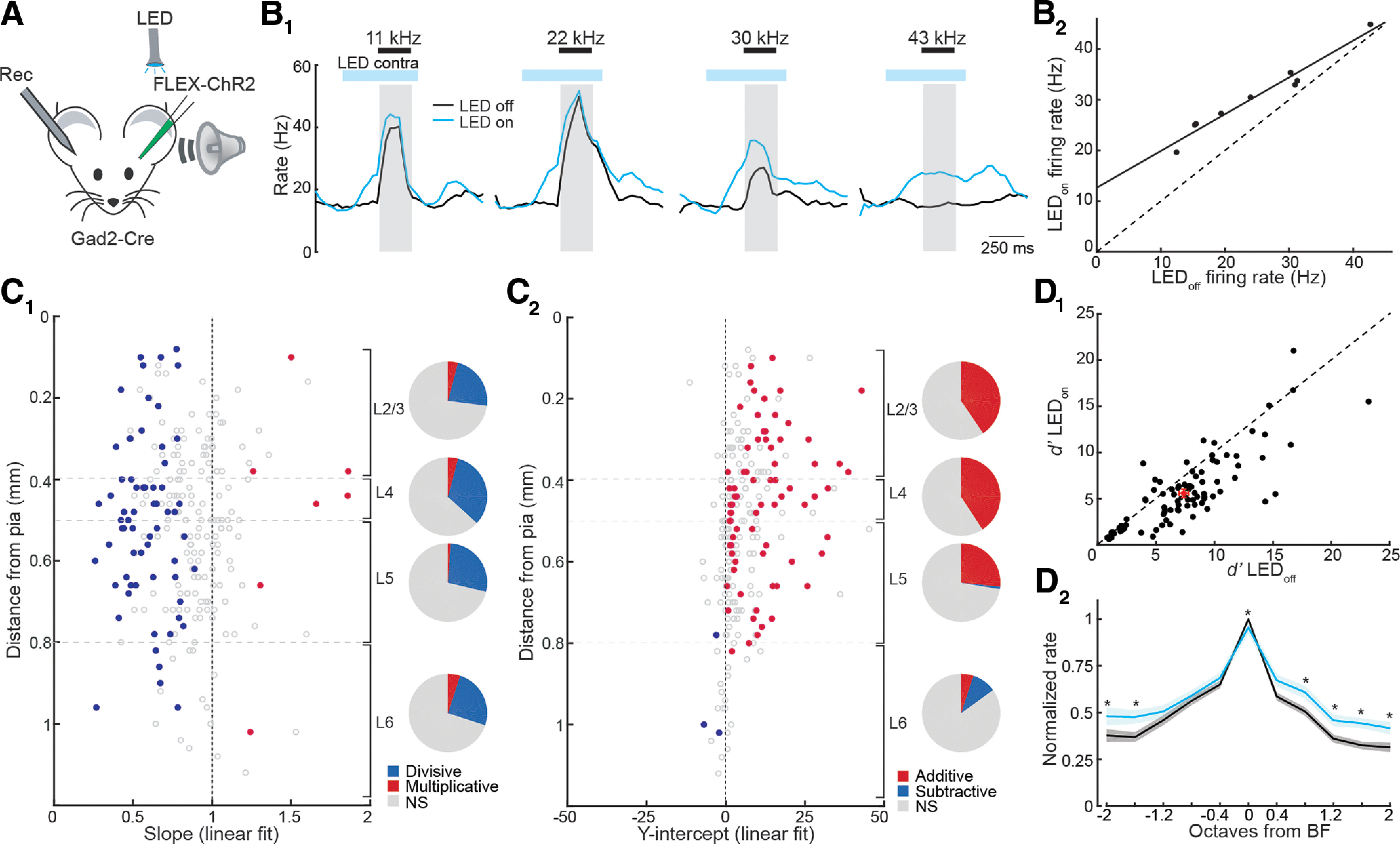
Silencing interhemispheric cortical input degrades the fidelity and frequency tuning of tone-evoked responses in A1. ***A***, Recording configuration. ***B***, Silencing contralateral A1 linearly modulates tone evoked activity via a combination of additive and divisive operations. ***B_1_***, Peristimulus time histograms (PSTHs) of tone-evoked responses from a representative RS unit to four frequencies (black bars) under control conditions (black line) and during contralateral silencing (blue line) on interleaved trials. Blue bars, LED pulse train. Gray, measurement windows for tone-evoked firing rate. ***B_2_***, Plot of firing rates during tones (*n* = 9 frequencies) with the LED on versus LED off of the cell in ***B_1_***. Line is linear fit: slope = 0.73, y-intercept = 12.63, *r*^2^ = 0.96. ***C***, Silencing callosal input exerts divisive and additive actions on tone-evoked activity across cortical layers. ***C_1_***, Slopes derived from linear fits to individual RS units with significant tone-evoked activity in each cortical layer. Blue circles, slope significantly <1. Red circles, slope significantly >1. Open circles, no significant change in slope. Pie charts represent fraction of cells in each layer with divisive (blue, slope <1), multiplicative (red, slope >1), or no significant effect (gray, NS). ***C_2_***, Y-intercepts derived from linear fits to same RS units in ***C_1_***. Blue circles, y-intercept significantly less than 0. Red circles, y-intercept significantly >0. Open circles, y-intercept not significantly different from 0. Pie charts represent fraction of cells in each layer with additive (red, y-intercept >0), subtractive (blue, y-intercept <0), or no significant effect (gray, NS). ***D_1_***, *d’* of RS units with LED off versus LED on shows that cortical silencing reduces response detectability. ***D_2_***, Cortical silencing “flattens” frequency tuning curves. Average tuning curves of RS units centered to their BF under control conditions (black) and during contralateral cortical silencing (blue). Asterisks indicate frequencies with significant difference (paired *t* test, Holm–Bonferroni corrected).

We next examined how silencing contralateral cortex modulates tone-evoked activity of RS cells in A1. The right ear was occluded and pure tones (nine log-spaced frequencies, 4–60 kHz, 250 ms, 60 dB) were delivered to the left ear during optogenetic silencing of the left hemisphere on interleaved trials (tone onset 250 ms following start of LED illumination; Fig. 4*A*). RS cells recorded from right A1 were frequency-tuned (Fig. 4*B*) such that particular frequencies drove strong firing (“preferred tones”) while others evoked weak responses (“non-preferred tones”). Interestingly, the effects of cortical silencing on RS cell activity were dependent on the strength of tone-evoked responses. Firing rates during non-preferred tones were enhanced by contralateral silencing, while firing evoked by preferred tones were largely unaffected or reduced (Fig. 4*B_1_*,*D_2_*). This effect could be described by a simple linear transformation: firing rates during tones with versus without LED-induced silencing could be fit by a line with a slope <1 and y-intercept > 0 (Fig. 4*B_2_*). In other words, removing callosal input had both an additive and divisive action on A1 tone responses. The effects of contralateral cortical silencing were uniformly divisive across all cortical layers (Fig. 4*C_1_*), while additive effects were prominent in all but L6 (Fig. 4*C_2_*). Together, these results suggest that callosal input normally regulates sound-evoked responses via multiplicative and subtractive effects.

Divisive/multiplicative operations exert gain control of neural responses while subtractive/additive operations modulate response fidelity via changes in variability associated with stimulus-independent (“background”) activity (Silver, 2010; Isaacson and Scanziani, 2011). Both the increase in spontaneous activity and additive effects on tone responses during contralateral cortical silencing suggest that callosal inputs enforce response fidelity. To address this possibility, we computed the discriminability index (*d’*; Materials and Methods), a measure of response reliability from signal detection theory (Tolhurst et al., 1983; Duguid et al., 2012; Sturgill and Isaacson, 2015) with and without contralateral cortical silencing. Optogenetic cortical inactivation significantly reduced the discriminability of tone-evoked activity (*d*′_LED-off_ = 7.37 ± 0.45, *d*′_LED-on_ = 5.58 ± 0.41, *n* = 124, *p *<* *0.001, *t* test; Fig. 4*D_1_*), indicating that callosal input normally serves to enhance the representation of tone responses relative to spontaneous activity in A1.

We examined how callosal input modulates the shape of frequency tuning curves by normalizing cell responses to their BF (tone eliciting strongest increase in firing) under control conditions. Silencing contralateral cortex caused a small decrease in the amplitude of responses at BF (*p* = 0.01, *t* test; Fig. 4*D_2_*), consistent with the divisive effect we observed on input-output relationships (Fig. 4*C*). However, because of its additive action, cortical silencing also increased responses to non-preferred frequencies. The net effect is thus a “flattening” of the population frequency tuning curve (Fig. 4*D_2_*). Thus, in addition to regulating response fidelity, callosal inputs normally play an important role in enforcing the sharpness of frequency tuning in A1.

### Data availability

All data discussed in the paper will be made available to readers on request.

## Discussion

We show that activating interhemispheric callosal projections can inhibit pyramidal cells in all layers of A1 in awake mice. These findings are consistent with slice recordings indicating that callosal inputs evoke strong feedforward inhibition of pyramidal cells in supragranular and infragranular layers. This feedforward inhibition likely reflects the recruitment of PV cells, which receive stronger callosal excitation than SOM or VIP cells in upper and lower cortical layers. In loss-of-function experiments, acute *in vivo* silencing of contralateral cortex increased pyramidal cell spontaneous activity in all but L6. Finally, we used tone-evoked activity to show that cortical silencing linearly transforms A1 input-output relationships via subtractive and divisive operations. This indicates that interhemispheric projections normally enhance the salience of tone representations (by regulating signal-to-noise ratio) and sharpen frequency tuning in A1.

It is well established that callosal inputs make direct excitatory connections onto cortical pyramidal cells (Karayannis et al., 2007; Petreanu et al., 2007; Lee et al., 2014, 2019; Rock and Apicella, 2015; Anastasiades et al., 2018) and drive disynaptic feedforward inhibition via contacts onto local GABAergic interneurons (Karayannis et al., 2007; Rock and Apicella, 2015; Anastasiades et al., 2018). Indeed, we found that brief activation of callosal fibers drives a biphasic increase and decrease in the firing of RS and FS cells in awake mice. Surprisingly, individual RS cells across all cortical layers were more likely to be inhibited than excited by callosal stimulation. In contrast, FS cells were more routinely activated, suggesting that the suppressive effects of callosal stimulation on RS cell firing are because of widespread PV cell-mediated feedforward inhibition. Consistent with this idea, brain slice recordings revealed that PV cells receive more callosal input than neighboring pyramidal cells or other interneuron subtypes and deep layer PV cells received ∼2× stronger input than L2/3 PV cells.

Previous studies in sensory cortical areas have used callosal sectioning (Payne et al., 1980; Engel et al., 1991) or reversible cortical cooling to probe the functional role of callosal inputs in anesthetized animals (Cerri et al., 2010; Schmidt et al., 2010; Carrasco et al., 2013, 2015; Wunderle et al., 2015). We show in awake mice that acute optogenetic silencing has heterogeneous effects on spontaneous activity: although a subset of RS cells shows a rapid and sustained decrease in activity, the majority of cells responded with a slow sustained increase in firing. The most straightforward interpretation of these results is that decreases in activity reflect the withdrawal of direct excitatory callosal input onto particular cells, while paradoxical increases in firing reflect indirect network effects. Increases in firing are most likely because of a reduction in inhibition provided by PV cells. Indeed, we observed that the spontaneous firing of deep layer PV cells was strongly suppressed during contralateral cortical silencing. This suggests that much of the tonic activity of deep layer PV cells is driven by interhemispheric input. Deep layer interneurons have recently been shown to project axons through all cortical layers toward the pia (Bortone et al., 2014; Frandolig et al., 2019). It is possible that interlaminar projections from deep layer PV interneurons mediate the indirect network effects underlying principal cell excitation following withdrawal of callosal input.

In contrast to previous work in auditory cortex of anesthetized animals (Carrasco et al., 2013, 2015), we did not observe a simple reduction in the strength of tone-evoked responses during contralateral silencing in the awake state. Rather, input-output plots of tone-evoked firing were linearly transformed in a divisive and additive fashion. Linear transformations (additive/subtractive and multiplicative/divisive) of sensory-evoked activity have routinely been observed across cortical areas when local circuits are perturbed (Atallah et al., 2012; Lee et al., 2012; Wilson et al., 2012; Sturgill and Isaacson, 2015; Phillips and Hasenstaub, 2016; Natan et al., 2017). Our findings of a mixture of divisive and additive operations presumably reflects the combination of the withdrawal of direct callosal excitatory input on pyramidal cells and layer specific reduction in feedforward inhibition. Higher spontaneous activity and stronger inhibition in the awake state are likely to underlie these differences (Haider et al., 2013; Kato et al., 2015). The actions of callosal inputs cannot be explained purely by a uniform modulation of PV-interneuron activity, since inactivation of PV-interneurons caused changes in principal neuron frequency tuning that were primarily additive and multiplicative (Seybold et al., 2015; Phillips and Hasenstaub, 2016). Differential callosal input to deep layer versus superficial layer PV cells could play a role in the effects on sensory coding we observe.

In addition to enhancing the discriminability of sound-evoked responses by maintaining a high signal-to-noise ratio, callosal inputs sharpen frequency tuning in A1. The functional impact of this interhemispheric modulation is different from that often reported in studies examining modulation by long-range cortical inputs. For example, somatosensory input can change tuning via a shift in preferred frequency (Gao and Suga, 2000; Ma and Suga, 2001), while olfactory input causes context specific modulation (Cohen et al., 2011). Inputs from the visual and motor systems can cause a uniform suppression of auditory responses that do not change frequency representations (Bizley et al., 2007; Kayser et al., 2008; Schneider et al., 2014, 2018). The findings in the current study are in agreement with previous studies indicating that interhemispheric connections modulate the specificity of sensory-evoked activity in visual (Hubel and Wiesel, 1967; Schmidt et al., 2010; Wunderle et al., 2015) and somatosensory cortex (Clarey et al., 1996). In future, it will be useful to determine how callosal input contributes to binaural cortical sound representations and auditory-directed behaviors such as sound localization and discrimination.

## Synthesis

Reviewing Editor: Christine Portfors, Washington State University Decisions are customarily a result of the Reviewing Editor and the peer reviewers coming together and discussing their recommendations until a consensus is reached. When revisions are invited, a fact-based synthesis statement explaining their decision and outlining what is needed to prepare a revision will be listed below. The following reviewer(s) agreed to reveal their identity: Khaleel Razak, Jeffrey Mellott.

The reviewers agree that the manuscript provides advances in the field by showing that callosal connections to auditory cortex increase neural frequency selectivity and signal to noise ratio. The methods are sophisticated and the data are strong. However, the reviewers agree that, as written, the manuscript does not provide clear enough justification or detailed enough methods to highlight the importance of the study. In particular, the methods are not described well enough for any other group to attempt to replicate the study. The reviewers agree that the main revision to the manuscript is in re-writing, not conducting new experiments.

Reviewer 1 comments:

In the manuscript, ‘Interhemispheric callosal projections sharpen frequency tuning and enforce

response fidelity in auditory cortex’, the authors describe the effect of activating or silencing auditory cortex (AC) of one hemisphere on responses in the contralateral side. They provide strong evidence using in vivo and in vitro preparations that the main effect of callosal activation in the auditory pathway is to increase, on average, excitation onto fast-spiking PV positive interneurons. This effectively reduces activity of pyramidal cells in the opposite hemisphere. The also inactivated the AC, and found an increase in spontaneous activity and a reduction in frequency tuning sharpness in the opposite cortex. The effect of callosal activation is stronger in PV, compared SOM and VIP cells. Overall, this paper presents relatively novel data, uses state-of-art methods, appropriate statistics and design and is well written. My comments are mostly minor as provided below.

1. Details in the methods section need to be improved. For example, there is no information on sound levels (e.g., in lines 136-137) used in this study. There needs to be an explanation of specific utility of each of the AAVs used in this study. I had to read the results to infer this information. The IACUC information is not properly provided. In many instances, it is mentioned that the mouse was anesthetized, but only once was isoflurane mentioned. The anesthetic and percent/dose has to be mentioned each time the procedure is mentioned. It is likely the mouse was anesthetized when the head bar was implanted, but this needs to be mentioned.

2. In methods or results, no verification is provided for the specificity of injections in P0-P2 mice in AC. This is in regards to the in vitro experiments. It would be useful to show if callosal axons are present in the contralateral AC and the layers/depths.

3. For in vitro experiments, it is mentioned, ‘Experiments were performed >2 weeks’. Provide actual range of days. Earlier in the methods, it is mentioned that slice experiments were performed in 3-5 week brains. >2 weeks is vague.

4. To compare the in vitro and in vivo results, it would be useful to know if PV/SOM/VIP cells are adult-like at 3-5 wks. Is anything known about development of callosal connections in AC pathways?

5. How were the neonatal mice anesthetized?

6. Line 127 - it is not clear why the cortex was covered with cyanoacrylate glue?

7. The mixed use of left/right AC and ipsilateral/contralateral cortex makes the methods difficult to read. The schematics in the figure are useful, but perhaps the methods writing can make this clear as well. For e.g., lines 131-132, why was the ear contralateral to the recording sites occluded?

8. Is the cyanoacrylate glue toxic used to occlude the ear an irritant to the ear canal? Was any sort of drug given to alleviate this?

9. Line 140 - rise/fall times, repetition rate?

10. Any information on penetrance of the LED based activation through cortical layers would be useful.

11. In the introduction and discussion, providing a comparative analysis of the cross-hemispheric auditory connections literature would improve the paper considerably. It is likely that callosal projections have different anatomical specificity and function across mammals. A carnivore with its high acuity binaural hearing is unlike to have the same function for callosal connections as a rodent with its lower sound localization accuracy. Indeed, the early studies of cats suggested that callosal connections connect EE (binaurally excited cells) on each side. Although it is mentioned that homotypic tonotopic regions may be connected in the mouse, it is not what is suggested by their results. A more nuanced comparison of the existing comparative AC callosal connections literature would improve the manuscript.

12. Similarly, a stronger explanation of the theory of ‘additive/divisive’ allusions of the results with appropriate references would be useful.

Reviewer 2 comments:

Assessment of the Manuscript:

The authors present data (with both in vitro, in vivo and optogenetic techniques ) showing that callosal projections between the auditory cortices excited fast spiking (inhibitory) cells and inhibited regular spiking (excitatory) cells. While there are a number of strong data sets within the manuscript, the major take home points concluded from the authors was that 1) callosal projections, in general, preferentially target PV expressing interneurons in deeper cortical layers and 2) silencing the callosal projections during tone-evoked activity could transform the input-output dynamic in auditory cortex leading to poorer frequency sharpening and poorer signal to noise ratios.

In general, the paper is well-written, the methods are sophisticated, the data are elegant, the statistical analysis is adequate and the manuscript is worthy of publication. However, there are a few moderate to minor concerns.

Moderate concern:

1) The title of the paper uses the term “auditory cortex” (which without additional improvements and/or addressing) I believe is the proper term to use.

However, the Significance, Results and Discussion routinely use “AI” or “primary auditory cortex”.

•While authors describe a method for identifying AI with intrinsic imaging, I feel that merely citing Kato et al., 2015 is not sufficient and that an image illustrating how AI was determined from intrinsic signaling should be used in Figure 1 of the manuscript so that the readers better understand exactly where the authors are drawing boundaries for AI. This particular concern goes hand and hand with a concern in which dashed lines are added to coronal sections in Figures 1 and 3 without any labels or reference in the figure legend. It also leaves reader curious as to how those lines, assuming they are meant to delineate AI, are achieved from intrinsic imaging.

•Then, for the in vitro work in the Methods, the area of interest is listed as auditory cortex, not primary; line 107. Though in the Results it is AI being referred to. This makes is confusing to compare results if the in vivo work was supposedly targeting AI while the in vitro work was just generally auditory cortex.

•Lines 187/188. The fact that label is seen at a low mag view covering the entire MG, at an A-P level in which most of the MBG area is likely to be non-lemniscal does not confirm the injections targeted AI. Why couldn’t most of the injection be in the anterior auditory field and still label the MGB in the same manner? In fact, I would argue, though it is hard to precisely determine due to the resolution of the figure, that the robust label in MG includes non-lemniscal nuclei/subdivisions and is likely indicative that the deposit for this particular case did encroach outside of AI and label portions of non-primary auditory cortex.

Taken together, and not to simplify the Authors’ efforts, it is curious why go through the extra steps of intrinsic signaling to identify AI when the use of landmarks and the superficial temporal vein was sufficient for the in vitro work and the use of a standard atlas could better explain how AI was identified in the coronal plane.

In the end, the Title should match the rest of the paper. If the Authors want the paper/title to be about primary auditory cortex rather than auditory cortex then they need to address the above concerns to make the Methods, Figures, figure legends and results a more cohesive AI story.

2) Lines 24/25: A noted Significance is that “Callosal projections make preferential input onto parvalbumin-expressing interneurons, particularly to those in deeper layers.” In the current state, it is unclear how this finding is different than what was already identified by Rock and Apicella, 2015. To the Authors credit, they do cite this paper in lines 63/64 for this very reason. It is just curious as to why it is listed in the Significance section if it has already been established.

3) Line 124: Cite or explain why the power of laser used was chosen in the experiments. And to what spatial extent was cortex silenced.

Minor concerns:

Lines 33/67/339/353: Carrasco is preceded with a first initial and then later with the full first name. Please correct.

Line 47: Please add a citation for the recent studies being referred.

Line 55: As the authors note, it is well known that the callosal projections from auditory cortex to auditory cortex are homotypic. However, this knowledge, at least what is cited is all from cats. There are a number of studies showing that in specific transgenic mouse lines, that the corpus callosum does not develop in a “normal” manner. If there are citations for the mouse corpus callosum organization and the callosal homotypic nature of the auditory cortices, it would helpful to cite.

Line 99: “and the skull above left AI identified by intrinsic signaling” I presume that the skull itself was not identified by intrinsic signaling.

There are some citations/sentences in the Results that are unnecessary or don’t belong.

Line 253: Atasoy et al., 2008; this should have been cited in the Methods.

Line 254: Kato et al, 2015

Line 289-296: All of these citations belong in either the Methods or Discussion. The paragraph would read more clearly if it was structured like the previous entries; e.g. “We next examined how divisive/multiplicative operations....” Introducing these concepts in the middle of the Results is odd rather than the Methods.

Figure 1 A1: it would be helpful for you to note the strain used as you did in Figure 3. It would also likely help if you note the strain in the text in lines 186/187, as you did with the other experiments/paragraph beginnings.

On a purely aesthetic note, setting up red and black to represent FS and RS, respectively, throughout the figures; only to have all the pie charts in red and a dark blue is distracting. Especially when all the figures have such small text and legends.

In Figure 2 A2 and Figure 3 B2, adding labels for the cortical layers for your recording site photomicrograph would greatly aid the reader.

Figure 3: It is unclear what the green lines represent; are they claiming that the callosal input only occurred in layers 2/3 and middle of 5? Or is it in between those 2 lines? Personally, the green lines are sort of unnecessary, but if they are to stay, the figure legend should give some explanation.

Lastly, this does not have to be addressed, but just an observation. It was somewhat surprising how little discussion was given to the layer V microcircuitry and its potential roles in auditory processing with the current findings. Nor was there much if any discussion on layer 6 cells even though in three of the experiments, regular spiking cells in layer 6 had differing properties than the rest of the layers. Additional commentary on these specific layer 6 properties and the known corticofugal and commissural circuitry of layer 6 would bolster the Discussion.
